# Glucocorticoid-Induced Leucine Zipper-Mediated TLR2 Downregulation Accounts for Reduced Neutrophil Activity Following Acute DEX Treatment

**DOI:** 10.3390/cells10092228

**Published:** 2021-08-28

**Authors:** Erika Ricci, Elena Roselletti, Marco Gentili, Samuele Sabbatini, Stefano Perito, Carlo Riccardi, Graziella Migliorati, Claudia Monari, Simona Ronchetti

**Affiliations:** 1 Department of Medicine and Surgery, Pharmacology Division, University of Perugia, 06132 Perugia, Italy; erika.ricci.1985@gmail.com (E.R.); marcogentili1988@hotmail.it (M.G.); carlo.riccardi@unipg.it (C.R.); graziella.migliorati@unipg.it (G.M.); 2 Department of Medicine and Surgery, Medical Microbiology Division, University of Perugia, 06132 Perugia, Italy; elenaro@hotmail.it (E.R.); samuele.sabbatini@gmail.com (S.S.); stefano.perito@unipg.it (S.P.); claudia.monari@unipg.it (C.M.)

**Keywords:** glucocorticoids, GILZ, TLR2, neutrophil

## Abstract

Glucocorticoids are the most powerful anti-inflammatory and immunosuppressive pharmacological drugs available, despite their adverse effects. Glucocorticoid-induced leucine zipper (GILZ) is a glucocorticoid-induced gene that shares several anti-inflammatory properties with glucocorticoids. Although immunosuppressive effects of glucocorticoids on neutrophils remain poorly understood, we previously demonstrated that GILZ suppresses neutrophil activation under glucocorticoid treatment. Here, we sought to explore the regulation of Toll-like receptor 2 (TLR2) by the synthetic glucocorticoid dexamethasone (DEX) on neutrophils and the associated GILZ involvement. Peripheral blood neutrophils were isolated from wild type and GILZ-knock-out (KO) mice. TLR2 was found to be downregulated by the in vivo administration of glucocorticoids in wild type but not in GILZ-KO neutrophils, suggesting the involvement of GILZ in TLR2 downregulation. Accordingly, the TLR2-associated anti-fungal activity of neutrophils was reduced by DEX treatment in wild type but not GILZ-KO neutrophils. Furthermore, GILZ did not interact with NF-κB but was found to bind with STAT5, a pivotal factor in the regulation of TLR2 expression. A similar modulation of TLR2 expression, impaired phagocytosis, and killing activity was observed in circulating human neutrophils treated in vitro with DEX. These results demonstrate that glucocorticoids reduce the ability of neutrophils to respond to infections by downregulating TLR2 via GILZ, thereby reducing critical functions.

## 1. Introduction

Glucocorticoid-induced leucine zipper (GILZ) is a gene rapidly transcribed by either endogenous or pharmacologically administered glucocorticoids [1,2,3,4,5]. Several studies have demonstrated that GILZ exerts anti-inflammatory activity, often acting as a mediator of glucocorticoid actions but without the glucocorticoid-derived side effects [6,7,8,9,10]. While almost ubiquitously expressed, GILZ is rapidly induced by glucocorticoids primarily in immune cells of the adaptive and innate system and regulates their cellular functions (e.g., activation, differentiation, and apoptosis) [3,11,12,13,14,15,16]. Within the cells of the innate immune system, few studies have investigated the function of GILZ in neutrophils. Our group has previously demonstrated that GILZ is the mediator of glucocorticoid-induced Annexin A1 gene transcription in neutrophils, one of the primary and first discovered mechanisms by which glucocorticoids exert their powerful anti-inflammatory effects [17]. The consequence of the induction of Annexin A1 by GILZ is reduced neutrophil migration in the inflammatory site, which prevents an excessive uncontrolled immune response and limits potential tissue damage due to exaggerated neutrophil infiltration [18]. Another related function of GILZ in neutrophils is the inhibition of proteins in the ROS and MAP kinase pathway systems, such as NOX2, p47phox, ERK, and p38 [19]. Thus, once GILZ is expressed, it can suppress neutrophil activation, further preventing excessive neutrophil activation. In this manner, GILZ functions as a brake with regards to the cellular functions of neutrophils, in response to inflammatory stimuli. Interestingly, GILZ can also promote apoptosis in neutrophils, indicating that it is involved in several aspects of the complex regulatory system of neutrophil life [20,21,22].

Inflammation is regulated via a negative feedback loop to the hypothalamic-pituitary-adrenal (HPA) axis, which leads to the release of circulating glucocorticoids. These hormones function to suppress the inflammatory mediators by acting on several molecular targets of immune cells. The pharmacological administration of glucocorticoids achieves the same purpose. Due to the importance of either reducing an inflammatory response that may persist and lead to detrimental effects or maintaining a functional immune protection during a glucocorticoid “storm”, it is important to study the regulation of pivotal regulatory molecules of immune cells, especially those of the innate immune system. In particular, both few and controversial studies are available regarding glucocorticoid-mediated regulation of the cellular components of neutrophils, such as Toll-like receptor 2 (TLR2). TLR2 belongs to a family of type I transmembrane proteins expressed on the surface of several immune cells that recognize a wide range of pathogen-associated molecular patterns (PAMPs) [23,24]. Once activated, TLRs initiate intra-signalling pathways that ultimately lead to the transcriptional regulation of pro-inflammatory genes, including cytokines (e.g., type I IFNs) [25,26,27,28]. TLR2 recognizes various microbial components, including zymosan, lipotheicoic acid, peptidoglycan, and lipoproteins, present in viruses and fungi [29,30,31,32]. Although the modulation of TLR2 by glucocorticoids has been studied in cells of the innate immune system other than neutrophils, the results have occasionally been contradictory. To date, no such studies have been conducted in neutrophils. Furthermore, the expression of TLRs may differ between mice and human cells (e.g., the absence of TLR2 expression in human T cells) [33,34].

The purpose of this study was to explore the modulation of TLR2 expression in peripheral resting neutrophils following glucocorticoid treatment, with particular attention paid to GILZ as a potential mediator of glucocorticoid-induced effects.

## 2. Materials and Methods

### 2.1. Media and Reagents

RPMI-1640, L-glutamine, and fetal calf serum (FCS) were purchased from MICROTECH SRL (Pozzuoli, Napoli, Italy). Phosphate buffered saline (PBS 1X) was purchased from Gibco BRL (Paisley, Scotland, UK). Lympholyte^®^ Cell Separation Media (Cedarlane, CL5020, Burlington, ON, Canada) was purchased from Euroclone, (Milan, Italy). Human recombinant GM-CSF was purchased from Miltenyi Biotec (Bologna, BO, Italy), and dexamethasone (DEX) was obtained from LFM, (Caronno Pertusella, VA, Italy). Sabouraud dextrose agar plus chloramphenicol, yeast extract, and peptone, dextrose anhydrous (YPD) agar, human serum type AB, and a penicillin-streptomycin solution were purchased from Sigma Aldrich (St. Louis, MO, USA). All reagents and media were negative for endotoxin.

### 2.2. Mice

Male C57BL/6 GILZ-KO and WT littermate mice (aged 8–12 weeks old) were generated as previously described [35]. All mice were housed under specific pathogen-free conditions and a 12-12-h light/dark cycle, receiving water and food ad libitum, in accordance with the Animals (Scientific Procedures) Act 1986 Amendment Regulations (SI 2012/3039) and the EU Directive 2010/63/EU and in compliance with the ARRIVE guidelines [36]. Five mice per group were used.

### 2.3. Isolation of Mouse and Human Neutrophils

Peripheral neutrophils were isolated from mice using a neutrophil isolation kit (Myltenyi Biotech 130-097-658). Human leukocytes were isolated from buffy coats using density gradient centrifugation on Lympholyte^®^ Cell Separation Media (Euroclone CL5020). The pellet containing neutrophils and erythrocytes was treated with hypotonic saline to lyse the erythrocytes. Neutrophils were collected by centrifugation and counted and adjusted to 1 × 10^6^/mL in RPMI + 10% FCS (complete medium). Hemacolor staining revealed that these cells were comprised of over 99% neutrophils by morphological evaluation. Trypan blue was used to evaluate the viability of these cells.

### 2.4. In Vivo and In Vitro Treatment

Mice were intraperitoneally injected with DEX (10 mg/kg) and sacrificed after 3 h. Blood was collected by an intracardiac puncture. Human neutrophils (1 × 10^6^/mL) were left untreated or treated with either human recombinant GM-CSF (50 ng/mL) (Miltenyi Biotec 130-093-862), DEX (10^−7^ M/mL), or a combination of GM-CSF (50 ng/mL) and DEX (10^−7^ M/mL) for 3 h at 37 °C +5% CO_2_ in complete medium [37]. Following this incubation, neutrophils were collected by centrifugation and analyzed successively, as described below.

### 2.5. Flow Cytometry Analysis of TLR2 Expression

Isolated neutrophils were washed in PBS +1% FCS and subsequently stained for 10 min at 4 °C with an anti-TLR2-FITC antibody (Myltenyi Biotech 130-099-016). Flow cytometry analysis was conducted on a Beckman Coulter EPICS-MCL and analyzed with FC Express analysis software.

### 2.6. Candida Albicans Strain and Culture

The origin and the characteristics of the highly virulent *C. albicans* strain (CA-6) have been described previously [38]. The cultures were maintained by serial passages on YPD agar. *Candida albicans* was cultured at 30 °C in yeast extract-peptone-dextrose (YPD) broth overnight under slight agitation. Yeast cells, grown to the stationary phase, were harvested from the overnight YPD culture and resuspended to a final concentration of 5 × 10^7^ cells/mL in YPD to induce yeast form [39]. Yeast cells were harvested by suspending a single colony in saline, followed by two washes, counting on a hemocytometer, and adjusting to the desired concentration.

### 2.7. Anti-Candida Activity of Mouse and Human Neutrophils

Neutrophils from mice treated with DEX (10 mg/kg) or from humans pre-treated as described above were washed and resuspended in RPMI 1640 at a concentration of 1 × 10^6^/mL. Neutrophil killing activity was determined with a CFU inhibition assay. Briefly, mouse or human neutrophils (10^5^ cells/100 μL) were added to CA-6 (10^4^ cells/100 μL) (pre-opsonized with HS at 10% in RPMI for 30 min at 37 °C and extensively washed after opsonization) in flat-bottom 96-well microtiter tissue culture plates and incubated in RPMI 1640 for 2 h at 37 °C plus 5% CO_2_. Following this incubation, the plates were vigorously shaken, and cells were lysed by adding Triton X-100 (0.1% in distilled water to a final concentration of 0.01%). Serial dilutions were prepared in distilled water from each well. The samples were then spread on Sabouraud dextrose agar plus chloramphenicol (50 μg/mL) in triplicate, and CFU values were evaluated after 24 h incubation at 37 °C. Control cultures consisted of CA-6 incubated in RPMI-1640 without effector cells. Candidacidal activity was expressed as the percentage of CFU inhibition according to the following formula: % of *C. albicans* inhibition growth = (1 − (CFU experimental/CFU of *Candida* cultured without cells) × 100) [40,41]. In selected experiments, the phagocytic capacity of human neutrophils (1 × 10^5^/200 μL), the cells were incubated with pre-opsonized CA-6 (2 × 10^5^/200 μL) in RPMI 1640 for 30 min, then collected by cytospin (700× *g* for 7 min) and stained with Hemacolor. Fungal cell internalization was expressed in accordance with the following formula: % of internalization = number of cells containing one or more fungal cells/100 cells counted [19].

### 2.8. Quantitative Real-Time PCR

Neutrophil RNA was isolated using a RNeasy+ Micro Kit (QIAGEN) and reverse-transcribed using the PrimesScript RT Reagent Kit with gDNA Eraser (Takara, Japan). Quantitative real time PCR was conducted using an ABI7300 Real Time Cycler PCR System (Applied Biosystem, Waltham, MA, USA), and the amplifications were performed using a Syber Green Gene Expression Master Mix (Applied Biosystems, Waltham, MA, USA). All reagents were purchased from Thermo Fisher Scientific (Waltham, MA, USA). GILZ was amplified as previously described [18], and β-actin was used as an internal control. All experiments were carried out in triplicate, and the ΔΔCt method was used to determine the level of target gene expression.

### 2.9. Immunofluorescence

Peripheral blood was collected from the mice after 3 h of in vivo DEX treatment, and neutrophils were isolated as described above. Murine neutrophils were cytospun onto a glass slide at 400 rpm for 4 min. Spotted cells were washed in phosphate-buffered saline (PBS) and permeabilized with an incubation in paraformaldehyde 4% for 20 min at room temperature. After washing three times in PBS, cells were blocked with blocking buffer containing 1% BSA, 10% horse serum 1h at room temperature. The slides were then incubated with a rabbit anti-STAT5 antibody (Elabscience, ESAP12240) or rabbit NF-κB (Cell Signaling) and goat anti-GILZ Ab (S-15; Santa Cruz Biotechnology, Dallas, TX, USA) overnight at 4 °C. After washing three times with PBS/Tween 0.1%, the slides were incubated for 1 h with secondary anti-rabbit-conjugated 488 and anti-goat-conjugated 555 antibodies (Alexafluor), and the nuclei were stained with diamidino-2-phenylindole (DAPI). After washing, the slides were mounted with a cover glass and analyzed using a Zeiss Axioplan fluorescence microscope.

### 2.10. In Situ Proximity Ligation Assay

The initial immune-staining steps were performed as described for immunofluorescence. After an overnight incubation with rabbit anti-STAT5 and goat anti-GILZ antibodies, anti-rabbit Minus and anti-goat Plus Proximity ligation assay (PLA) probes were added to a blocking buffer in accordance with the manufacturer’s instructions. All of the following steps were performed with Detection Reagents Red (Sigma-Aldrich, St. Louis, MO, USA), according to the manufacturer’s instructions. After the final washes, the slides were mounted with a cover glass and the detection of interaction signals was analyzed using a Zeiss Axioplan fluorescence microscope.

### 2.11. Statistical Analysis

Statistical analysis was performed using Prism 6.0 software (GraphPad, San Diego, CA, USA). A two-tailed unpaired Student’s *t*-test was used for statistical comparisons after the assessment of data distribution by the Saphiro-Wilk normality test (* *p* < 0.05; ** *p* < 0.01; *** *p* < 0.001).

## 3. Results

### 3.1. TLR2 Expression on Murine Neutrophils

**Figure 1:**
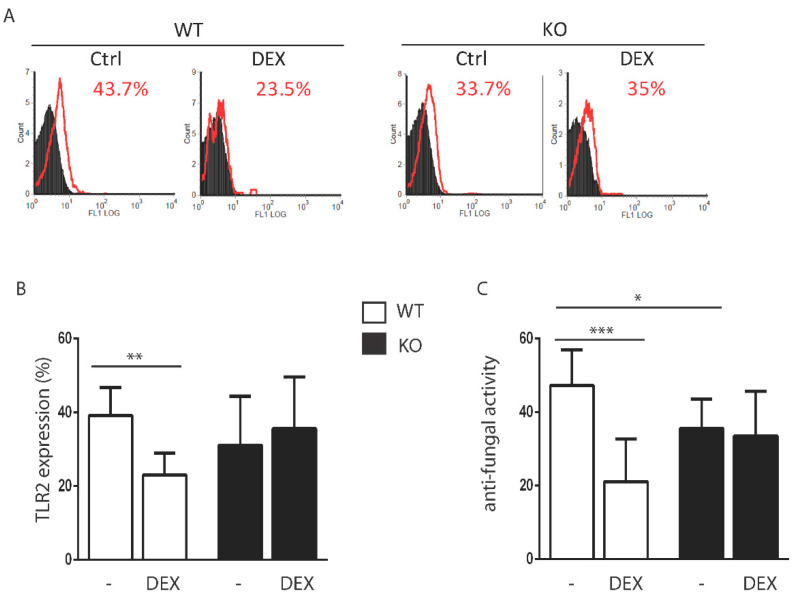
TLR2 expression and neutrophil function was regulated by glucocorticoids via GILZ. (**A**). Flow cytometry evaluation of Toll-like receptor 2 (TLR2) expression in neutrophils isolated from wild type (WT) and GILZ-Knock-out (KO) mice following 3 h treatment with dexamethasone (DEX). Isolated neutrophils were analysed for TLR2 expression (red line) as the percentage of positive cells; the black histogram represents the isotypic control. A representative experiment is shown. (**B**). TLR2 expression on neutrophils. (**C**). Anti-fungal activity of WT and KO neutrophils, as evaluated in vitro against *Candida albicans*. Mean value of three independent experiments ± SD. *N* = 2–3/group. * *p* < 0.05; ** *p* < 0.01; *** *p* < 0.001.

Since GILZ is one of the most important early-induced genes by glucocorticoids, we aimed to define the role of glucocorticoids in the regulation of TLR2 in neutrophils in wild type (WT) and GILZ knock-out (KO) mice. We administered the synthetic glucocorticoid DEX to WT and KO mice and evaluated the level of TLR2 expression on isolated peripheral neutrophils by flow cytometry after 3 h. Figure 1A,B shows that treatment with DEX downregulated TLR2 in WT but not in the KO mice, suggesting that GILZ was involved in glucocorticoid-mediated downregulation of TLR2. Since TLR2 is upregulated following exposure to several microorganisms and also regulates neutrophil functionality, a reduction in its expression can account for the anti-inflammatory effect of glucocorticoids on neutrophils as well as a reduction in the sentinel function of these cells. Therefore, we explored the anti-fungal activity of peripheral neutrophils in the same experimental settings. We found that DEX reduced the candidacidal activity of neutrophils in WT neutrophils, but not in KO cells from DEX-treated mice (Figure 1C), suggesting that GILZ was involved in this function, which is linked to TLR2 expression. Interestingly, peripheral resting neutrophils from the KO mice exhibited a spontaneous reduction in anti-*Candida* activity when compared to WT mice.

### 3.2. GILZ-Mediated Regulation of TLR2 in Mouse Neutrophils

**Figure 2:**
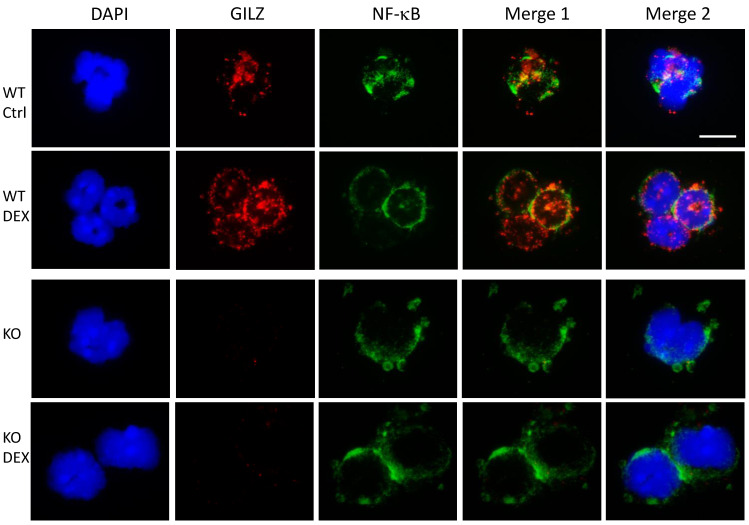
GILZ and NF-κB staining in mouse neutrophils does not overlap. Isolated peripheral WT and KO neutrophils were spotted on slides after 3h in vivo DEX treatment. Nuclei were stained with diamidino-2-phenylindole (DAPI). Cytoplasmic nuclear factor-κB (NF-κB) (green) and GILZ (red) staining did not show overlapping spots (yellow, merge 1) in untreated cells (upper panels) or in DEX-treated cells (middle panels). Overlapping staining of NF-κB, GILZ, and DAPI is shown in Merge 2. GILZ staining was absent in the KO neutrophils (lower panels). Scale bar: 10 µm. All experiments were repeated three times. The results of a representative experiment are presented.

To investigate the mechanism by which GILZ controls TLR2 expression, we first analysed the transcription factor NF-κB, which is a known GILZ-interacting protein, whose binding sites have been identified in the promoter of mouse TLR2 [11,42,43,44,45,46]. Thus, we sought to find a potential interaction between GILZ and NF-κB in neutrophils under our experimental conditions that could account for the observed reduction of TLR2 expression. To this end, we treated WT and KO mice with an intraperitoneal injection of DEX and isolated neutrophils from the peripheral blood after 3 h. We performed an immunofluorescence assay for NF-κB localization. Figure 2 shows that NF-κB was localized in the cytoplasm as expected (green fluorescence), in both WT and KO cells, but not in all cells, and never found in the nucleus, indicating no activation of this factor in any of ourexperimental conditions. GILZ was expressed in the cytoplasm, and increased expression was observed following DEX treatment, as expected [18]. Surprisingly, GILZ staining (red fluorescence) did not overlap with NF-κB staining (green fluorescence) in both the untreated and DEX-treated mice.

**Figure 3:**
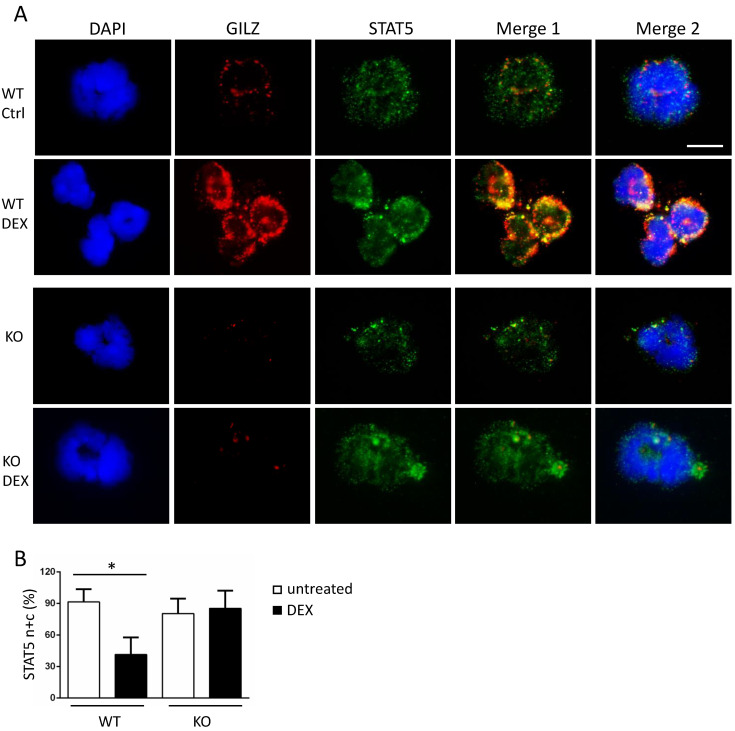
GILZ and STAT5 staining overlaps in mouse neutrophils. (**A**) Isolated peripheral WT and KO neutrophils were spotted on slides after 3 h of in vivo DEX treatment. The nuclei were stained with DAPI. Cytoplasmic and nuclear STAT5 (green) and cytoplasmic GILZ (red) staining showed overlapping spots (yellow, Merge 1) in DEX-treated cells (middle panels). Overlapping staining of STAT5, GILZ, and DAPI is shown in Merge 2. GILZ staining was absent in KO neutrophils (lower panels). (**B**) Cells with both cytoplasmic and nuclear (n + c) STAT5 staining were counted and expressed as a percentage of total cell number. Values represent mean ± SD. Scale bar, 10 µm. All experiments were repeated three times. The results of a representative experiment are presented. * *p* < 0.05.

**Figure 4:**
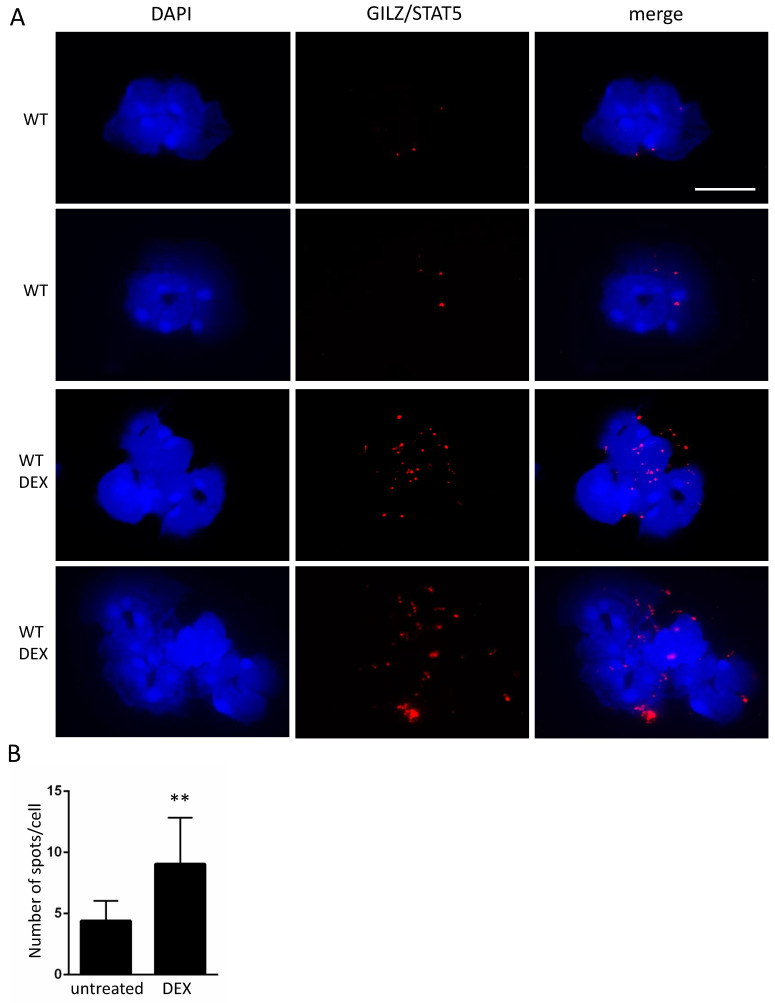
GILZ and STAT5 interact in DEX-treated neutrophils. (**A**) An in-situ proximity ligation assay in isolated peripheral WT and KO neutrophils of in vivo DEX-treated mice. Red spots indicate a GILZ/STAT5 interaction: low number of spots were observed in the WT untreated mice (two upper rows of panels), whereas a higher number of spots were identified in DEX-treated cells (two lower rows of panels). (**B**) Statistical analysis of the spot number was performed using ICY free software. Two fields of representative stainings are shown (WT and WT+DEX). Values represent the means ± SD of more than 20 fields. Scale bar: 10 µm. ** *p* < 0.01.

We next analyzed STAT5 as another important transcription factor involved in the regulation of TLR2 expression, under the same experimental conditions [46]. The immunofluorescence of STAT5 in Figure 3A reveals that STAT5 was expressed in all peripheral neutrophils (green fluorescence), in both the nucleus and cytoplasm, whereas GILZ (red fluorescence) was expressed only in the cytoplasm, as expected. Both GILZ and STAT5 expression was enhanced following DEX treatment, with both of them located in the cytoplasm. Cells expressing STAT5 in both cytoplasm and nucleus were counted over total cell number (Fugure 3B). A reduced percentage of DEX-treated WT cells with STAT5 staining in both cytoplasm and nucleus was observed, suggesting STAT5 localization predominantly in the cytoplasm. Importantly, STAT5 and GILZ expression overlapped in the yellow stained spots following in vivo DEX treatment, suggesting that the two proteins are extremely close and may potentially interact. To demonstrate this newly identified STAT5/GILZ potential interaction, we performed an “in situ Proximity ligation assay”, a technique that is used to prove an existing interaction between two proteins. Both positive and negative controls were used, as shown in Supplementary Figure S1. Figure 4 shows the appearance of red dots in the neutrophils of DEX-treated mice, indicating that there is a physical interaction between GILZ and STAT5. A few sporadic spots are also detectable in untreated cells. The total counts of spots in Figure 4B demonstrate that the GILZ-STAT5 interaction was significantly increased by DEX treatment.

### 3.3. TLR2 Modulation by Glucocorticoids in Human Neutrophils

**Figure 5:**
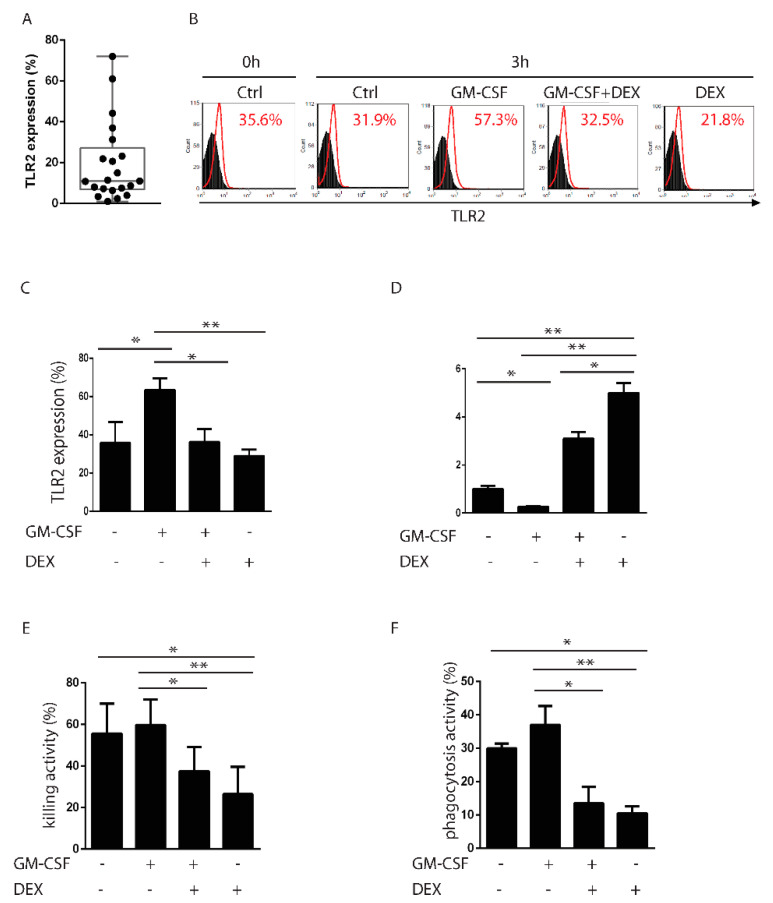
DEX downregulates TLR2 in human neutrophils. (**A**) Flow cytometry evaluation of TLR2 expression on human neutrophils (PMNs) isolated from buffy coats (*n* = 21). (**B**) In vitro treatment with the granulocyte-macrophage colony-stimulating factor (GM-CSF) upregulates TLR2 after 3 h (percentage of total cells), whereas DEX downregulates TLR2, either alone or in combination with GM-CSF. One representative staining is shown. (**C**) Summary and mean values of TLR2 expression as described in B. Values represent the mean ± SD of three independent experiments. (**D**) Quantitative RT-PCR of GILZ expression in PMNs under the same experimental conditions as described in (**A**). (**E**,**F**) Killing activity and phagocytosis, respectively, of PMNs treated in vitro with GM-CSF or DEX, either alone or in combination, or left untreated for 3 h. Values represent the mean ± SD of three independent experiments. * *p* < 0.05; ** *p* < 0.01.

Due to some discrepancies in TLR2 expression and transcriptional regulation between mouse and human neutrophils, we next sought to explore whether glucocorticoids could reduce TLR2 expression in polymorphonuclear neutrophils (PMNs) as observed in mouse cells. To this end, we first evaluated TLR2 expression on buffy coat-derived PMNs. As shown in Figure 5A, flow cytometry analysis revealed a substantial variability in TLR2 expression at basal levels in human PMNs, which is in line with previously published data [47]. To avoid any biased results associated with the baseline level of TLR2 expression, we activated PMNs with GM-CSF, which is known to upregulate TLR2 on human cells [37]. Therefore, PMNs were treated with GM-CSF, DEX, or a combination of GM-CSF plus DEX or were left untreated for 3 h. Our results showed that DEX downregulated the basal levels of TLR2 when used as single treatment but was also able to counteract the upregulation of TLR2 induced by GM-CSF (Figure 5B, representative experiment, and C, summary of all the experiments). In the same cells, we evaluated GILZ mRNA expression by real-time PCR and found elevated levels when TLR2 was downregulated, suggesting that GILZ upregulation by DEX might contribute to TLR2 downregulation (Figure 5D). Next, we analyzed the functionality of DEX-treated PMNs. Under the same experimental conditions, the killing activity of these cells was found to be impaired following DEX treatment and limited by DEX in the cells co-treated with GM-CSF (Figure 5E). We also evaluated the phagocytic activity of PMNs against *C. albicans* and found similar results, with an impairment in phagocytosis in the presence of DEX, when treated alone or co-treated with GM-CSF (Figure 5F).

## 4. Discussion

The anti-inflammatory and immunosuppressive properties of glucocorticoids as both stress hormones and drugs have long been widely known. In particular, immunosuppression by glucocorticoids is differentially regulated in blood cells. Neutrophils are the only immune cells in which glucocorticoids can prolong survival, which represents one of the mechanisms contributing to glucocorticoid-induced neutrophilia [48,49,50]. Glucocorticoid-mediated protection of neutrophils has been intended as a primary defence strategy against invading microbes [51]. In addition, glucocorticoids have been shown to modulate various molecular pathways in neutrophils, such that the resulting effect may be either pro- or anti-inflammatory [21,52]. One of the unexplored pathways is linked to TLR2, a pivotal receptor involved in the recognition of both microbial and viral components. TLR2 regulation by glucocorticoids has been shown to be somewhat controversial in different cell types. Therefore, due to the importance of TLR2 in primary host defence, the purpose of this study was to explore the mechanism of glucocorticoid-mediated control of TLR2 expression in neutrophils, in the presence or absence of GILZ, a crucial early glucocorticoid-induced gene. Our results demonstrated that TLR2 was downregulated by DEX in WT neutrophils, whereas no effect was observed in KO cells, in which DEX did not alter the level of TLR2 surface expression. Thus, GILZ is clearly involved in DEX-mediated TLR2 downregulation, since GILZ is basally expressed and over-expressed by DEX in neutrophils, as was previously demonstrated by our group [18]. The finding of DEX-mediated downregulation of TLR2 in neutrophils is in contradiction with that of studies conducted in other cells. As an example, glucocorticoid treatment upregulates TLR2 in dendritic cells, thereby impairing dendritic cell maturation. Moreover, in macrophages, glucocorticoid treatment can either increase TLR2 expression to release soluble TLR2 as an anti-inflammatory tool or decrease it to reduce macrophage activation [53,54,55]. Specifically, while TLR2 upregulation by glucocorticoids has been defined as quite “paradoxical” in the context of their anti-inflammatory effects, this effect can be considered to be linked to a positive feedback loop that appears to exist between TLR2 expression and glucocorticoid secretion in the adrenals. Indeed, TLR2-KO mice display reduced corticosterone levels [24,56]. In neutrophils, TLR2 expression is required for host antimicrobial defences [57,58,59]. Our data support this role since the anti-fungal activity was impaired in WT neutrophils treated with DEX but not in KO cells. This finding suggests a role for GILZ in the modulation of these functions, which is likely to reduce TLR2 expression. Similar to mouse neutrophils, DEX treatment reduced TLR2 expression on human PMNs, impairing both the associated phagocytosis and killing activities [60]. DEX was also able to prevent GM-CSF-dependent upregulation of TLR2, suggesting that any glucocorticoid-based therapy may impair neutrophil activity, reducing the sentinel function of neutrophils. Therefore, the concept of neutrophil protection by glucocorticoids should be revised or, at least, re-considered. We do not exclude additional mechanisms other than TLR2 regulation in the control of neutrophil functions, during glucocorticoid treatment, which must be deeply analyzed. This study explored some of the main neutrophil functions in which GILZ plays a role, but others (i.e., Netosis), known to be linked to TLR2 expression, deserve to be investigated as well [61].

Furthermore, we previously demonstrated that GILZ expression counteracts neutrophil activation [19]. Here, we found that GILZ expression was downregulated following GM-CSF treatment in PMNs, which was counteracted by DEX co-treatment, with opposed expression of GILZ over time to that of TLR2. This observation strengthens the GILZ-dependent regulation of TLR2 expression during glucocorticoid treatment and is in line with other studies in which TNF-α-induced expression of TLR2 was found to be enhanced by silencing GILZ in HUVEC cells [52]. Once again, GILZ proves to play an anti-inflammatory role by downregulating TLR2, the expression of which can be increased by pro-inflammatory cytokines [37].

GILZ heterodimerizes with partner proteins, primarily transcription factors, thus regulating gene transcription [6]. Therefore, we sought to identify possible partner proteins of GILZ involved in the regulation of TLR2 transcription. The binding sites for NF-κB and STAT5 have been previously found in the TLR2 promoter and are required for the induction of TLR2 gene expression [46,62]. NF-κB has been largely recognized as a partner protein of GILZ, whose binding elicits several anti-inflammatory effects by preventing its transcriptional regulation [43,63,64,65,66]. Moreover, GILZ- NF-κB binding is a mechanism by which GILZ exerts several functions, depending on the cell system used and the context in which inflammation is studied. While we were not able to identify any overlapping staining between NF-κB and GILZ, we could detect overlapping staining and an interaction with STAT5. Here, for the first time, we demonstrate the interaction of GILZ with STAT5, through which GILZ prevents STAT5-derived transcriptional upregulation of TLR2. It has been previously demonstrated that there is a functional interaction between the glucocorticoid receptor (GR) and STAT5 in the induction of the TLR2 gene in an epithelial cell line, under combined treatment with DEX and TNFα treatment, which is absent following DEX alone [62,67]. Our findings suggest that GILZ is a mediator of the glucocorticoid-mediated regulation of TLR2 through STAT5, adding a piece of information in the puzzling molecular gene regulation by glucocorticoids in neutrophils.

Another important issue to consider is the presence of GRE in the TLR2 promoter. It has been previously demonstrated that the presence of GRE-like elements in the TLR2 promoter alone are insufficient to induce glucocorticoid regulation of TLR2 expression [62]. This study proposes an indirect mechanism through GILZ, by which glucocorticoids regulate the transcription of the TLR2 gene, thus controlling and reducing neutrophil activity. On the other hand, GILZ could also inhibit TLR2-derived intracellular signalling, since NF-κB is activated downstream of the TLR2 signalling pathway, which further reduces neutrophil activity [24].

In conclusion, neutrophils remain the only immune cells protected by glucocorticoids from undergoing cell death, thus conferring a first-line host defence mechanism protection following pathogen attack. We present evidence that glucocorticoids cause a downregulation in the level of TLR2 expression, thereby contributing to an impairment in a main neutrophil function, i.e. pathogen recognition. For the first time, we demonstrate that GILZ, by interacting with STAT5, functions as an anti-inflammatory agent by reducing neutrophil activity, with a consequent reduction in pathogen recognition and potential major risk of exposure to pathogen-derived infection. It is clinically important to consider that a pharmacological acute treatment with glucocorticoids can prevent neutrophils from executing their defense role by this newly described TLR2-dependent mechanism.

## Data Availability

The data presented in this study are available on request from the corresponding author.

## Supplementary Materials

The following are available online at www.mdpi.com/article/10.3390/cells10092228/s1, Figure S1: In situ proximity ligation assay (PLA).
