# Supplementary material for: Glucocorticoid-Induced Leucine Zipper-Mediated TLR2 Downregulation Accounts for Reduced Neutrophil Activity Following Acute DEX Treatment
Source: Cells. 2021 Aug 28;10(9):2228. doi: 10.3390/cells10092228 (PMC8472062; doi:10.3390/cells10092228)
Supplement: Supplementary file 1 [file cells-10-02228-s001.zip › cells-1347893-supplementary.pdf]

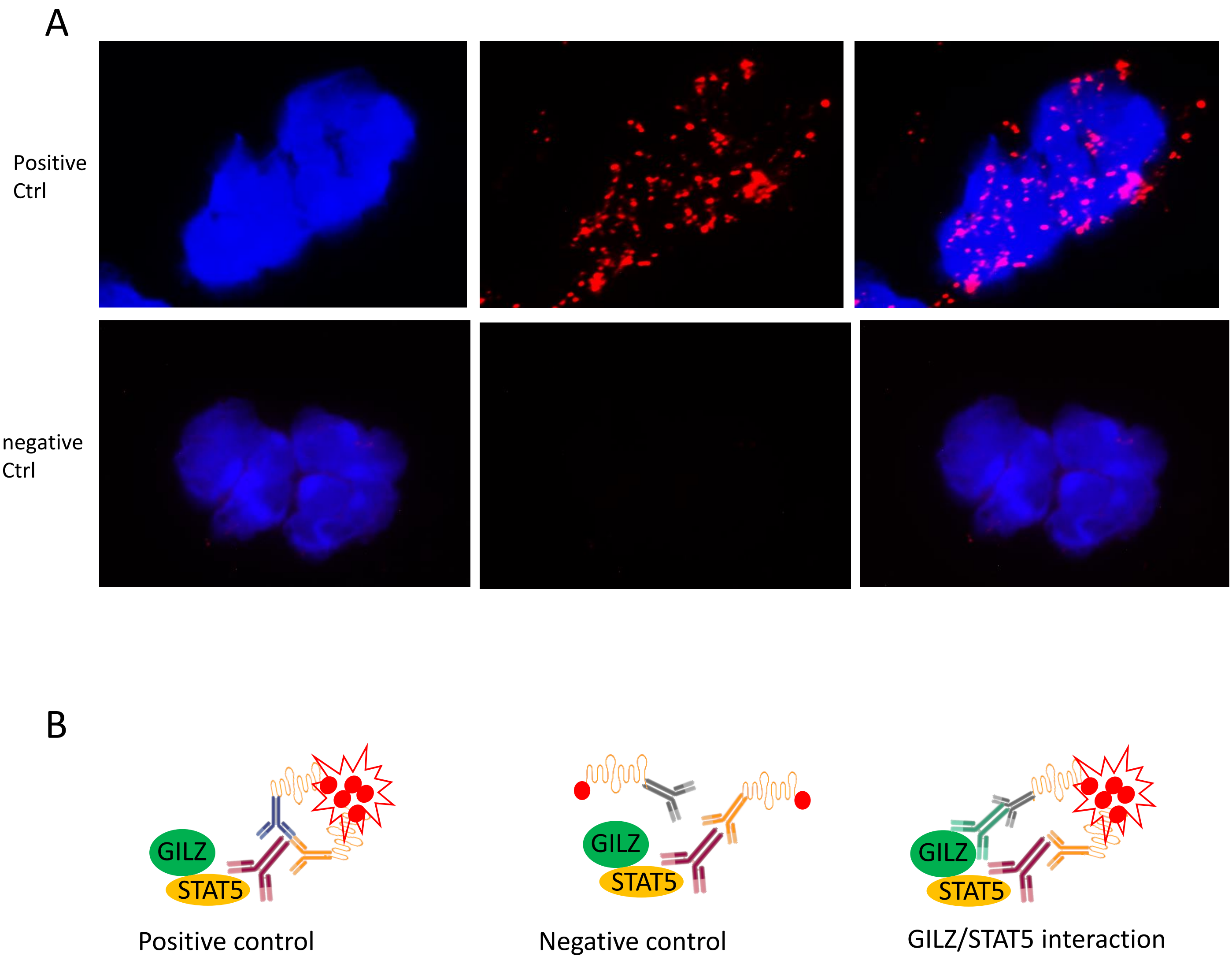

Figure S1. In situ proximity ligation assay (PLA): A. positive and negative controls (Ctrl) in untreated PMNs, using an anti-STAT5 antibody. B. Schematic rapresentation of the technique: Minus and Plus anti-rabbit PLA probes were used against the same anti-STAT5 Ab in the positive control, detecting STAT5 staining. Plus anti-goat and Minus anti-rabbit PLA probes were used in the negative control in which only anti-STAT5 Ab was added. GILZ and STAT5 interaction can be detected as depicted in figure, by using both antibodies with Plus anti-goat and Minus anti-rabbit PLA probes.

Figure S1
